# Is Ultra-High Temperature Processed Milk Safe in Terms of Heterocyclic Aromatic Amines?

**DOI:** 10.3390/foods10061247

**Published:** 2021-05-31

**Authors:** Fatih Oz, Emel Oz, Eyad Aoudeh, A. M. Abd El-Aty, Maomao Zeng, Theodoros Varzakas

**Affiliations:** 1 Department of Food Engineering, Faculty of Agriculture, Ataturk University, Erzurum 25240, Turkey; emel.oz@atauni.edu.tr (E.O.); eiadaudi@gmail.com (E.A.); 2 Department of Medical Pharmacology, Medical Faculty, Ataturk University, Erzurum 25240, Turkey; abdelaty44@hotmail.com; 3 Department of Pharmacology, Faculty of Veterinary Medicine, Cairo University, Giza 12211, Egypt; 4 State Key Laboratory of Food Science and Technology, Jiangnan University, Wuxi 214122, China; mmzeng@jiangnan.edu; 5 International Joint Laboratory on Food Safety, Jiangnan University, Wuxi 214122, China; 6 Department of Food Science and Technology, University of Peloponnese, Antikalamos, 24100 Kalamata, Greece

**Keywords:** UHT milk, heterocyclic amines, protein-fortified milk, lactose-free milk, growing up milk

## Abstract

Herein, the presence of heterocyclic aromatic amines (HAAs) in 24 different commercial ultra-high temperature processed (UHT) milk types was investigated. The dry matter and pH values of the samples were also determined. The milk types showed significant differences (*p* < 0.01) regarding the dry matter, pH values, and individual HAAs and total HAAs. The milk sample dry matter and pH values were in the range of 8.56–13.92% and 6.66–6.91, respectively. The growing up milk samples had the highest dry matter and pH values. While no significant correlation between the total HAAs and dry matter was found, a negative correlation (*p* < 0.01) between the total HAAs and pH value was determined. Among the tested HAAs, five compounds, (IQx (up to 0.06 ng), IQ (up to 0.10 ng), MeIQx (up to 0.55 ng), MeIQ (up to 1.97 ng), and PhIP (up to 0.39 ng)) were quantified in the samples. The average total HAAs of the samples ranged from 0.13 to 0.67 ng; however, one milk sample (200 mL) contained between 10.10 and 53.35 ng total HAAs. Therefore, it was shown that protein fortification and lactose hydrolysis substantially increased the formation of HAAs in UHT milk.

## 1. Introduction

Milk is a nutrient-dense food supplying all the essential components for growth and development. It contains water (87%), readily digestible fats (3–4%), high biological value proteins (3.5%), lactose (5%), minerals (1.2%), and vitamins (0.1%), in addition to many other minor constituents that have a positive effect on human health [1,2,3]. Therefore, milk and dairy products play a vital role in a balanced and healthy diet. Adequate intake of milk and dairy products, particularly during childhood, might have beneficial effects on health and reduce the risk of osteoporosis, hypertension, and obesity during adulthood [4,5].

In contrast, some studies showed that milk intake was associated with an increased risk of several diseases, such as cardiovascular disease, cancer, and diabetes, due to its high content of saturated fatty acids [1,4,6]. Over the past few years, changes have occurred in dairy products to satisfy consumer requirements, preferences, and needs. Presently, different types of ultra-high temperature processed (UHT) milk, such as growing up milk, lactose-free semi-skimmed milk, protein-fortified milk, whole milk, organic milk, skimmed milk, and semi-skimmed milk, are often found in markets.

Ultra-high temperature processed (UHT) milk is produced by processing raw milk at 135–150 °C for a few seconds (1–5 s) to ensure the destruction of pathogenic microorganisms and heat-resistant spores and the inactivation of indigenous enzymes that may cause the milk to spoil. However, heat treatment may be associated with many negative changes in milk, such as protein denaturation, vitamin degradation, a brown color, and off-flavor formation [7]. 

During the thermal processing of milk, the Maillard reaction occurs, leading to advanced Maillard reaction products [6,8]. Hence, some undesirable compounds can also be found in heat-treated milk [6,9]. Several authors have extensively investigated the Maillard reaction products formed in heat-treated milk. These have been used in assessing thermal processed milk’s quality and shelf-life examination [8,10,11,12].

Heterocyclic aromatic amines (HAAs) are chemical compounds produced during the thermal treatment of protein-rich food [13,14]. So far, approximately 30 HAAs have been isolated and identified in thermally processed food products. Among them, eight have been considered as possible carcinogens for humans (class 2B) and one as a probable human carcinogen (Class 2A) by the International Agency for Research on Cancer [15]. There was a positive association found between HAA exposure and different types of cancer [14]. In addition, HAAs are more mutagenic than aflatoxin B_1_ (over 100-fold) and benzo[a]pyrene (over 2000-fold). 

Depending on the formation potentials, HAAs are classified into two major groups, thermic and pyrolytic. Thermic HAAs are generated from a reaction between free amino acids, creatine/creatinine, and hexoses (Maillard reaction) during the thermal processing of foods over the range of 150–300 °C, forming heterocyclic pyridines and pyrazines, which undergo further transformation yielding imidazoquinoxalines. The second group was produced due to the pyrolysis of amino acids and proteins at temperatures higher than 300 °C [14,16]. 

Presently, HAAs have been detected in diverse types of foods, including meat (up to 480 ng/g) [14], roasted coffee (up to 2930 ng/g) [17,18], alcohol beverages (up to 42.30 ng/g) [18], cheese (up to 44.42 ng/g) [19], toasted bread (up to 164.2 ng/g) [18], eggs (up to 2.06 ng/g) [20] as well as the breast milk of healthy mothers (up to 1 ng/mL) [21]. Up to the authors’ best knowledge, the presence of HAAs in UHT milk has not yet been fully elucidated. Further, it is unknown whether this modified milk is safe in terms of mutagenic and/or carcinogenic HAAs. Hence, 24 different UHT milk samples were inspected regarding heterocyclic aromatic amine contents and their dry matter content and pH values.

## 2. Materials and Methods

### 2.1. Chemicals and Reagents

All chemicals and solvents used for HAA analysis were HPLC-grade and procured from Merck Co. (Darmstadt, Germany). Ultra-pure water was obtained from a Milli-Q water purification system (Millipore, Direct Q^@^3 UV). Oasis MCX extraction cartridges (3 cm^3^/60 mg) were secured from Waters (Milford, MA, USA). The standards of HAAs including IQx, IQ, MeIQx, MeIQ, 7,8-DiMeIQx, 4,8-DiMeIQx, 4,7,8-TriMeIQx, PhIP, AαC, and MeAαC were acquired from Toronto Research Chemicals (Downsview, Ontario, ON, Canada). 4,7,8-TriMeIQx was used as an internal standard. Standard stock solutions in methanol were prepared as described by Oz [22].

### 2.2. Sample Collection

A total of 24 × 3 different commercial UHT milk samples were purchased from local markets in Erzurum (Turkey). Samples were grouped into nine different types (according to their contents): growing up milk (n = 4), lactose free semi-skimmed milk (n = 2), protein-fortified coffee-flavored milk (n = 1), protein-fortified cocoa-flavored milk (n = 2), protein-fortified vanilla-flavored milk (n = 2), whole milk (n = 5), organic milk (n = 2), skimmed milk (n = 2), and semi-skimmed milk (n = 4). 

Specific consideration was given to purchase different batch numbers for each replicate (three replicates, the total number of milk samples was 72). The nutrition fact labels of milk samples according to the manufacturer declaration are given in Table 1. Raw milk used in recovery analyses was also brought from a local market in Erzurum, Turkey. All milk samples were analyzed before their expiry dates and within the same day of opening.

### 2.3. Dry Matter and pH

The dry matter of UHT milk samples was performed following the method described by AOAC Official Method [23]. The milk sample (3 g) was accurately weighed into a flat bottom dish and dried in an oven at 102 °C until constant weights were achieved. The pH value was measured using a pH-meter (Seven Compact-S220, Greifensee, Switzerland) at 20 °C.

### 2.4. Sample Preparation for Determination of Heterocyclic Aromatic Amine

The heterocyclic aromatic amines (HAAs) were extracted according to the method described by Oz et al. [24] with some modifications. The milk sample (2.5 mL) was weighed in a beaker, to which 12 mL 1 M NaOH was added and stirred for an hour. Afterward, 10 g Extrelut NT packaging material (Merck, Darmstadt, Germany) was added, mixed thoroughly, and then transferred to the Extrelut column. The extraction was performed using 75 mL ethyl acetate and passed through a coupled and previously conditioned Oasis MCX cartridge. 

The cartridge was washed with hydrochloric acid and methanol. After that, the analytes were eluted with 2 mL methanol: ammonia (19:1, *v/v*) and filtered through a 0.45 mm filter. The eluent was evaporated to dryness at 45 °C, and the residues were redissolved in 100 μL methanol (with internal standard, 1 ng/mL) before HPLC analysis. The individual and total HAA amounts were expressed as ng in 2.5 mL milk. Most of the purchased milk samples were sold in bottles of 200 mL capacity. Therefore, the portion size was expressed as ng in 200 mL (2.5 × 80).

### 2.5. HPLC Analysis

The HAAs were analyzed using HPLC (Thermo Ultimate 3000, Thermo Scientific, Waltham, MA, USA) equipped with an autosampler (WPS-3000), a pump (LPG-3400SD), and a diode array detector (DAD-3000). A reversed-phase analytical column (Acclaim^TM^ 120 C_18_, 3 μm, 4.6 × 150 mm) from Tosoh Bioscience GmbH (Stuttgart, Germany) was used for separation, and the column temperature was maintained at 35 °C. A gradient program was applied using a mobile phase of methanol: acetonitrile: deionized water: acetic acid (8:14:76:2, *v/v/v/v*) as a solvent A (pH = 5.0 adjusted with ammonium hydroxide (25%)); and acetonitrile as a solvent B. HAAs were identified by their retention times and quantified using an external calibration curve.

### 2.6. Method Validation

The method was validated (single laboratory validation) in terms of the limit of detection (LOD), the limit of quantification (LOQ), linearity, recovery, and precision. The calibration curves were established from known concentrations. The correlation coefficients (*R*^2^) were evaluated by regression analysis. The recovery rates were determined using the standard addition method, applied by adding a specific volume of known concentration (three different concentrations) of mixed standards into raw milk samples and processed as mentioned above [25]. The intra-day and inter-day precisions were determined by analyzing mixed HAAs at various concentrations in duplicate daily and repeated for four consecutive days. We also spiked raw milk samples with three different concentrations of mixed HAAs, followed by applying extraction procedures in duplicate daily for two consecutive days.

### 2.7. Statistical Analyses

The samples were analyzed in triplicate (24 × 3 = 72 samples). Triplicates of each sample belonging to the same brand and the same type were interpreted as a whole. The experimental data were subjected to variance analysis, and Duncan’s multiple range test was used to compare between the means. The differences were considered statically significant at *p* < 0.05. Statistical calculations were performed using the SPSS version 20 IBM SPSS statistics package program. Principal Component Analysis (PCA) was conducted by SIMCA-P + 14.1 software (UMETRICS, Umea, Sweden).

## 3. Results

### 3.1. Dry Matter and pH Values of Milk Samples

The dry matter contents and pH values of the milk samples are presented in Table 2. The milk types showed significant differences (*p* < 0.01) in terms of the dry matter, which ranged between 8.56% and 13.92%. The highest dry matter was detected in the growing up milk samples, attributed to high carbohydrate and fat contents (Table 1). On the other hand, the lowest percentage of dry matter was recorded in skimmed milk. This finding could be ascribed to the very low and moderate fat and carbohydrate contents, respectively. The pH values ranged between 6.66 and 6.91 (Table 2). 

A significant difference (*p* < 0.01) was also noticed between the pH values of different milk types. Like dry matter, the growing up milk samples had the highest pH values; however, the lowest values were recorded in the protein-fortified cocoa-flavored milk samples. The differences in pH values could be assigned to the differences in their contents. This study’s achieved dry matter and pH values aligned with those recorded by others [26,27,28].

In general, the dry matter and pH values of milk samples showed significant (*p* < 0.01) differences in terms of the brand (Supplementary Table S1). Regarding the same type of milk samples obtained from different brands, the dry matter content showed either significant (*p* < 0.01) differences in most types or no significant differences in some types: growing up milk, lactose-free-semi-skimmed, and organic milk in terms of the brand. On the other hand, significant (*p* < 0.05) differences were observed between them in all types of pH values. These differences between brands could be associated with the raw material quality, process conditions, ingredient quality, and storage conditions.

### 3.2. Method Validation

The limits of detection (LOD) and quantification (LOQ) of the nine HAAs were estimated based on 3 and 10 times the signal/noise ratios, respectively. The LOD, LOQ, linear equation, and *R*^2^ are shown in Table 3. The LOD and LOQ ranged from 0.004 to 0.025 ng/g and 0.013 to 0.085 ng/g. The *R*^2^ of the tested HAAs was <0.9996. The relative standard deviation (RSD%) < 11% (Table 4). Table 5 shows the RSD% and recovery (%) of the tested HAAs in raw milk fortified at three different concentrations. As shown in the Table 5, the RSD values were <15%, and the recoveries ranged from 72.76–96.23%. The obtained values were consistent with the findings obtained by others [22,29,30].

### 3.3. Heterocyclic Aromatic Amines Content of Milk Samples

Herein, 72 different milk samples were analyzed for their HAA contents, including IQx, IQ, MeIQx, MeIQ, 7,8-DiMeIQx, 4,8-DiMeIOx, PhIP, AαC, and MeAαC. Four individual HAAs (7,8-DiMeIQx, 4,8-DiMeIOx, AαC, and MeAαC) were not detected in any of the tested samples. The milk type showed a significant difference (*p* < 0.01) in terms of the individual HAAs and total HAA amount. The minimum, maximum, and average levels of individual HAAs, total HAAs, and the proportions in different milk samples are given in Table 6. The IQx content ranged from non-detectable to 0.06 ng in 6 out of 72 analyzed milk samples (8%). 

The highest average IQx content (0.05 ng) was found in protein-fortified coffee-flavored milk samples. In addition, IQx was also detected in the growing up and protein-fortified cocoa-flavored milk samples; however, its level was lower than the LOQ value. IQ was detected in 32 samples (44%), including all types of milk, at a range of non-detectable to 0.10 ng. As with the IQx compound, the highest average IQ content (0.09 ng) was measured in protein-fortified coffee-flavored milk samples. Up to 0.03 ng IQ was determined in protein-fortified cocoa-flavored milk; however, its level in other samples was lower than LOQ. MeIQx was detected in 57 samples (79%), including all milk samples, except protein-fortified coffee-flavored milk samples. Its amount ranged from non-detectable to 0.55 ng. 

Interestingly, the highest average MeIQx content (0.41 ng) was recorded in organic milk. MeIQx is one of the predominant HAAs in the current research and accounted for 14–100% of the total HAA amount. MeIQ, the other predominant HAA herein, was detected in 21 samples (29%). Its amount ranged from non-detectable to 1.97 ng. The highest average MeIQ content (0.43 ng) was displayed in protein-fortified vanilla-flavored milk samples. PhIP was detected in 17 samples (24%), and its concentration ranged from non-detectable to 0.39 ng. 

The highest average PhIP content (0.19 ng) was determined in protein-fortified coffee-flavored milk samples. MeIQx and PhIP are detected in growing up milk samples and can lead to health problems in toddlers. This is because MeIQx, MeIQ, and PhIP are possibly carcinogenic (Class 2A) to humans, as documented by IARC [15]. The average total HAA contents in milk samples ranged between 0.13 to 0.67 ng (Table 6). The highest average total HAA levels were detected in protein-fortified vanilla-flavored milk, whereas the lowest was recorded in growing-up milk samples (Table 6). 

The higher levels of total HAAs in protein-fortified vanilla-flavored milk samples could be because this milk type is fortified with protein and hydrolyzed lactose, which increased the precursors of HAAs. On the other hand, the lower total HAAs in protein-fortified coffee- and cocoa-flavored milk samples could be attributed to the antioxidant effect of coffee and cocoa, which play an essential role in inhibiting HAAs formation. In addition, it should be noted that different components in the milk samples could also play different roles in the formation or inhibition of HAAs in milk. 

Even if the total HAA content in growing up milk was low in the present study, it is noteworthy that toddlers consume this milk type. The daily consumption of milk is recommended by most dietary guidelines with different serving sizes between 125 to 250 mL [3]. Most of the milk types in Turkey are sold in bottles with a 200 mL capacity. Considering this serving size, the investigated milk types in this research contained 10.10–53.35 ng average total HAAs (Table 6).

It is difficult to compare the results of this study with others because none have been published regarding the formation of IQx, IQ, MeIQx, MeIQ, 7,8-DiMeIQx 4,8-DiMeIOx, PhIP, AαC, and MeAαC in UHT milk. Pouzou et al. [31] estimated the daily exposure of the U. S. population to HAAs to be 565.3 ng/day. Another study calculated the average dietary intake of HAAs to be 103 ng/d in Germany [32]. At the same time, in Croatian women, the mean exposure to total HAAs was estimated to be 4.43 ng/kg BW per day [33]. Almost all previous studies considered meat as the principal source of exposure to HAAs. However, as compiled in Table 6, the diverse types of UHT milk also have a considerable amount of total HAAs. Therefore, consuming such types of milk might contribute to the daily exposure to HAAs.

The samples (n = 72) tested in this study varied significantly in terms of their chemical composition (protein, carbohydrate, fat, and lactose). Therefore, to facilitate the interpretation of the results and determine the effect of the milk samples’ contents on the formation of HAAs, the samples were grouped into subgroups according to their protein, carbohydrate, fat, and lactose contents. In terms of their protein contents, the samples were grouped as low (less than 2.4%; n = 4), medium (between 2.5% and 4%; n = 15), and high (higher than 4%; n = 5) protein contents. 

As shown in Table 7, significant differences were noticed between subgroups regarding the total HAA content and HAA consumption per portion. The protein contents of the milk samples had a significant effect (*p* < 0.01) on the HAA contents. The greater the protein content, the more the total HAA content. As known, heterocyclic amines are formed during the thermal processing of protein-rich foods, forming the reaction of creatine and/or creatinine, sugars, and free amino acids. Since free amino acids are the major precursors of HAAs, the heating of food proteins may result in the formation of HAAs [14,16,34].

According to the carbohydrate contents, samples were grouped into low (less than 5%; n = 13), medium (between 5% and 5.9%; n = 6), and high (higher than 6%; n = 5) carbohydrate contents. As shown in Table 7, no significant differences were noticed between the milk samples regarding the total HAA level and portion. It was stated by Oz and Kaya [14] and Alaejos and Afonso [16] that a certain level of sugar could promote the formation of HAAs. 

In contrast, the addition of excessive amounts of sugar vs. other precursors (amino acids and creatine) tended to inhibit HAA formation. Further, it was demonstrated that sugar was not mandatory for forming imidazoquinoline type HAAs (IQ-type, Aminoimidazoazoarenes, and AIAs) in model systems. In the light of the obtained results, we may conclude that the impacts of the protein content were more influential than the carbohydrate content on the formation of the total HAAs in milk.

Samples were also grouped into skimmed (less than 0.5%; n = 7), semi-skimmed (between 1% and 2%; n = 6), and whole milk (higher than 2%; n = 11) based on their fat contents. We found no significant differences between the milk samples grouped according to their fat contents regarding the total HAA level and portion (Table 7). The role of dietary fat on the formation of HAAs is not fully understood. However, research stated that the optimum fat level for the maximum formation of HAAs was 10% in minced meat [35]. 

In addition, Nilsson et al. [36] found that fats could promote the formation of HAAs only if the processing temperature was higher than 200 °C. In sum, the reason why milk fat does not have a significant effect on the formation of HAAs could be ascribed to the fact that milk has less fat compared to meats, and the temperature during UHT processing is below the effective temperature of fats.

Regarding lactose, the samples were divided into lactose-free (n = 7) and lactose (n = 17). Our results showed that lactose hydrolysis led to significant increases in the total HAA content of UHT milk samples. The total HAA content (0.40 ± 0.38 ng/2.5 mL milk) of lactose-free milk was significantly higher than that of (not hydrolyzed; 0.24 ± 0.18 ng/2.5 mL milk) samples with lactose. The hydrolysis of lactose into its constituents (glucose and galactose) resulted in increasing the reduction in sugar molarity. In addition, glucose and galactose substantially promoted the Maillard reaction compared with lactose in protein–sugar models [37,38,39].

The Maillard reaction proceeded at a greater rate in protein–galactose and protein–glucose models than in an equivalent protein–lactose model during heating [37,38]. At the same time, lactose hydrolysis doubles the reducing sugar molarity in milk, thus, favoring the Maillard reaction [39]. Consequently, lactose-hydrolyzed and heat-treated milk is more prone to non-enzymatic browning than regular milk [40,41].

### 3.4. Correlation

With further data analysis, a positive correlation between the total HAAs and both MeIQx (r = 0.42; *p* < 0.01) and MeIQ (r = 0.76; *p* < 0.01), which are the dominant HAAs in the milk samples of the present study. The pH value had a moderate negative correlation with the total HAA levels (r = −0.43; *p* < 0.01) and MeIQ content (r = −0.54; *p* < 0.01). In line with our results, Zamora et al. [42] reported a linear decrease of MeIQ formation when the pH increased from 6.5 to 9. Similarly, a negative correlation was observed between the level of HAAs and the pH value in various heat-treated foods [22,43]. 

**Figure 1:**
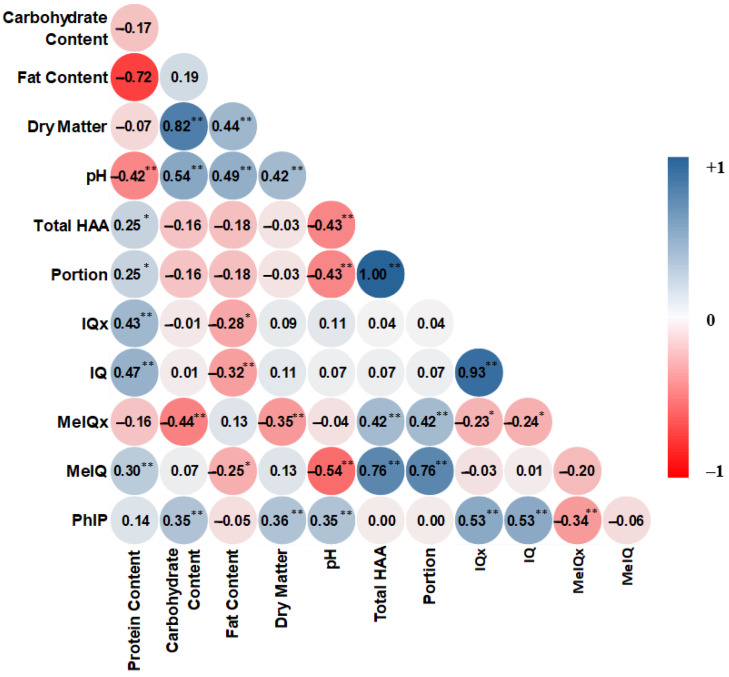
Pearson’s correlation coefficient among different chemical and physiochemical properties of UHT milks. (**: *p* < 0.01; *: *p* < 0.05).

No significant correlations were observed between the total HAAs and carbohydrate content, while the correlation was significant between the carbohydrate content and both MeIQx (r = −0.44; *p* < 0.01) and PhIP (r = 0.35; *p* < 0.01). We assumed that the higher content of protein (which led to an increase in free amino acids as one of the protein degradation products) could be associated with higher HAA contents. Herein, a positive correlation was found between the protein content and both the total HAAs (r = 0.25; *p* < 0.05) and some individual HAAs, including IQx (r = 0.43; *p* < 0.01), IQ (r = 0.47; *p* < 0.01), and MeIQ (r = 0.30; *p* < 0.01) (Figure 1).

### 3.5. Discrimination of UHT Milk Samples Using PCA

**Figure 2:**
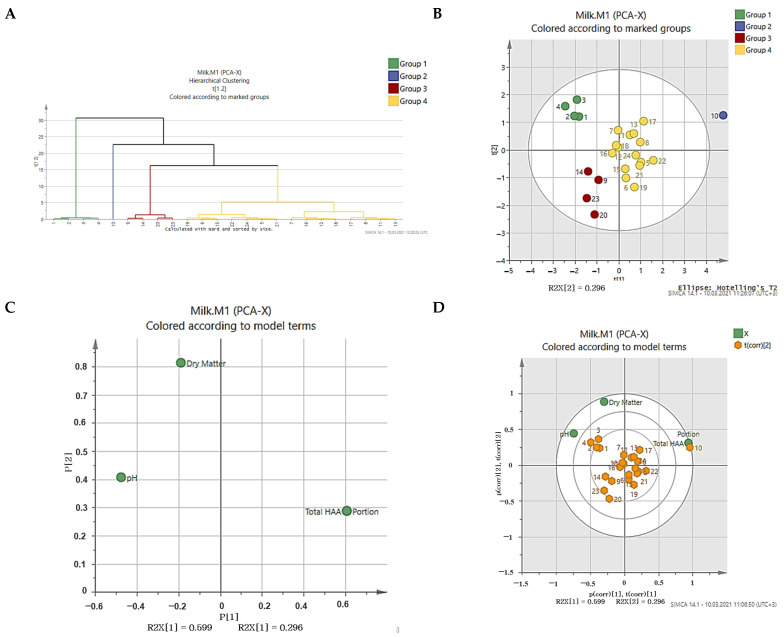
Dendrogram (**A**), score scatter plot (**B**), loading scatter plot (**C**), and biplot (**D**) of the principal component analysis (PCA) (PC1 versus PC2) for the attributes in the milk samples.

PCA was applied to illustrate the differences between milk samples from the dry matter content, pH value, total HAA content, and HAA consumption per portion. The hierarchical clustering, score scatterplot, loading scatter plot, and biplot of samples are shown in Figure 2A–D, respectively. The first two principal components (PC1 = 59.9% and PC2 = 29.6%) accounted for 89.5% of the variance. As a result of the analysis, it was possible to classify the milk samples into four main groups (Figure 2A,B). The milk sample (protein-added, lactose-free, and vanilla-flavored milk) coded with number 10 was outside the circle, which indicates that this milk sample was significantly different from the other samples. 

The milk samples (growing up milk) coded with numbers 1, 2, 3, and 4 were allocated in the same group, which denotes that these milk samples had similar traits. While the milk samples coded with 9, 14, 20, and 23 were assigned in the same group, all remaining samples were allocated together (Figure 2B). The total HAA content and HAA consumption per portion were clustered at the right side of the plot, while the dry matter and pH were clustered at the left side of the plot (Figure 2C). This result showed that the total HAA content and HAA consumption per portion were negatively correlated with the dry matter and pH. In contrast, there was a positive correlation between the dry matter and pH (Figure 2C). 

The total HAA content and HAA consumption per portion were located at the left part of PC1, indicating that the milk samples (protein-added, lactose-free, and vanilla-flavored milk) coded with number 10 contained higher levels of total HAAs, while the milk samples coded with 9, 14, 20, and 23 contained lower levels of total HAAs. On the other hand, milk samples (growing up milk) coded with the numbers 1, 2, 3, and 4 had a higher dry matter and pH (Figure 2D).

## 4. Conclusions

This is the first study to inspect UHT milk in terms of heterocyclic aromatic amines. The results revealed that all types of UHT milk contained heterocyclic aromatic amines. MeIQx and MeIQ were the predominant HAAs in almost all milk types. From the health perspective, the consumption of 200 mL milk may include total HAAs at 10.10–53.35 ng level. Protein fortification and lactose hydrolysis were related to a significant increase in the formation of HAAs in UHT milk. Further study is, therefore, needed to investigate the influence of UHT parameters on the formation of HAAs. Care should also be given to the positive association found between HAA exposure and different types of cancer and mutagenicity effects.

## Figures and Tables

**Table 1:** The nutrition facts label of milk samples according to the manufacturer’s declaration.

Brand	Type	n	Protein Content	Carbohydrate Content	Sugar Content	Fat Content
A	Growing up milk	3	2.1	7.8	5.3	2.8
B	Growing up milk	3	2.1	7.5	4.9	2.9
C	Growing up milk	3	1.9	8.1	7.9	3
D	Growing up milk	3	2.3	8	7.4	3
B	Lactose free-semi skimmed	3	2.9	4.7	4.7	1.5
D	Lactose free-semi skimmed	3	3	4.7	4.7	1.5
B	Protein-fortified coffee-flavored milk ^•^	3	6	5.4	5.4	0.1
D	Protein-fortified cocoa-flavored milk ^•^	3	5.2	6.5	5.6	0.3
B	Protein-fortified cocoa-flavored milk ^•^	3	6	5	5	0.1
D	Protein-fortified vanilla-flavored milk ^•^	3	5.2	5.8	5.8	0.3
B	Protein-fortified vanilla-flavored milk ^•^	3	6	5.8	5.8	0.1
E	Whole milk	3	3	5.6	3.9	3
B	Whole milk	3	3	4.7	4.7	3
D	Whole milk	3	3	4.5	4.5	3.3
F	Whole milk	3	3	4.6	4.6	2.5
G	Whole milk	3	2.9	4.8	3.4	3.3
B	Organic milk	3	3	4.7	4.7	3
D	Organic milk	3	3	4.6	4.6	3
B	Skimmed milk	3	2.9	4.7	4.7	0.1
D	Skimmed milk	3	3.1	5	5	0.1
A	Semi-skimmed milk	3	2.9	4.7	2.7	1.7
H	Semi-skimmed milk	3	2.9	4.7	4.7	1.5
F	Semi-skimmed milk	3	3	4.7	4.7	1.5
G	Semi-skimmed milk	3	2.9	4.8	3.5	1.5

^•^: skimmed and lactose-free; n: replicate.

**Table 2:** The dry matter contents (%) and pH values of milk samples (mean ± SD).

Type	Dry Matter (%)	pH
Growing up milk	13.92 ± 0.27 ^a^	6.91 ± 0.05 ^a^
Lactose free - semi skimmed	9.69 ± 0.18 ^f^	6.74 ± 0.03 ^d,e^
Protein-fortified coffee-flavored milk^•^	11.92 ± 0.02 ^b^	6.82 ± 0.01 ^b^
Protein-fortified cocoa-flavored milk^•^	11.86 ± 1.42 ^b^	6.66 ± 0.08 ^g^
Protein-fortified vanilla-flavored milk^•^	11.63 ± 0.57 ^c^	6.69 ± 0.16 ^f^
Whole milk	11.19 ± 0.54 ^d^	6.76 ± 0.03^d^
Organic milk	11.57 ± 0.16 ^c^	6.80 ± 0.03 ^c^
Skimmed milk	8.56 ± 0.35 ^g^	6.73 ± 0.02 ^e^
Semi skimmed milk	9.93 ± 0.21 ^e^	6.74 ± 0.03 ^d,e^
Sig.	**	**

SD: standard deviation; Sig: significance; **: *p* < 0.01; ns: not significant; ^a–g^: means with different letters in the same column are significantly different (*p <* 0.05). ^•^: all protein-fortified milk samples were skimmed and lactose-free.

**Table 3:** The limits of detection (LOD) and quantitation (LOQ), linear equation, and regression coefficients (*R*^2^) for different heterocyclic aromatic amines.

HAA	LOD (ng/g)	LOQ (ng/g)	Linear Equation	*R* ^2^
IQx	0.004	0.013	y = 2.3138x − 0.1007	0.9996
IQ	0.009	0.029	y = 1.0272x + 0.0226	0.9999
MeIQx	0.024	0.081	y = 1.6509x − 0.0574	0.9998
MeIQ	0.014	0.047	y = 1.3127x − 0.0060	0.9999
7,8-DiMeIQx	0.005	0.018	y = 2.5318x − 0.0799	0.9999
4,8-DiMeIOx	0.008	0.025	y = 1.9723x − 0.0627	0.9997
PhIP	0.025	0.085	y = 0.1777x − 0.0032	0.9999
AαC	0.012	0.039	y = 0.4733x − 0.0151	0.9999
MeAαC	0.010	0.035	y = 0.3473x − 0.0499	0.9999

**Table 4:** The relative standard deviation (RSD%) of different heterocyclic aromatic amines in blank milk analyzed at six different concentrations.

HAAs	Concentration (ng/mL)	IQx	IQ	MeIQx	MeIQ	7,8-DiMeIQx	4,8-DiMeIQx	PhIP	AαC	MeAαC
Intra-day RSD % (n = 8)	10	3.14	3.23	2.16	1.71	1.97	1.78	1.43	0.88	0.65
	7.5	1.71	1.97	0.99	1.78	1.24	1.72	1.63	1.28	1.21
	5	0.91	0.84	1.04	1.31	1.1	1.04	1.12	0.88	0.77
	2.5	1.37	1.72	1.45	1.54	0.91	2.11	1.16	1.28	1.40
	1	1.47	1.97	1.60	1.07	1.78	3.01	1.05	1.11	3.11
	0.5	1.18	1.56	1.32	1.36	1.04	1.46	2.34	1.91	2.38
Inter-day RSD % (n = 8)	10	8.37	3.98	7.84	5.01	7.91	6.79	7.98	6.23	4.34
	7.5	9.92	4.33	8.91	5.62	8.87	7.48	9.00	7.53	4.40
	5	9.21	3.38	8.27	4.29	8.04	7.79	8.13	6.39	2.98
	2.5	10.02	5.12	9.47	6.66	8.87	8.52	9.27	6.93	3.16
	1	9.75	4.18	9.14	4.42	8.99	8.42	9.4	6.89	4.63
	0.5	10.94	5.57	9.87	5.96	9.95	7.68	9.57	5.18	7.48

**Table 5:** The relative standard deviation (RSD%) and recovery (%) of different heterocyclic aromatic amines in raw milk spiked at three different concentrations.

HAAs	Concentration (ng/mL)	Intra-Day (n = 4)	Inter-Day (n = 4)	Recovery (%)
		RSD%	RSD%	
IQx	10	1.01	2.04	89.78
	5	3.90	6.65	91.36
	1	1.09	1.25	86.91
IQ	10	6.39	6.38	84.28
	5	2.73	7.02	87.47
	1	1.88	1.83	84.41
MeIQx	10	2.73	2.73	93.64
	5	3.75	6.15	94.59
	1	2.07	2.07	89.80
MeIQ	10	5.72	5.72	94.01
	5	3.27	6.71	96.23
	2.5	2.59	2.92	89.82
7,8-DiMeIQx	10	3.24	3.24	91.84
	5	3.46	5.57	93.26
	1	1.54	1.54	87.51
4,8-DiMeIQx	10	3.04	5.19	92.05
	5	3.52	3.52	90.70
	1	0.59	2.58	84.65
PhIP	10	4.03	5.41	94.40
	5	1.97	1.97	96.20
	1	0.80	0.80	91.96
AαC	10	2.21	2.15	85.65
	5	12.23	9.54	87.33
	1	9.04	9.04	82.79
MeAαC	10	3.16	3.16	85.65
	5	14.44	12.17	82.92
	1	12.48	12.47	72.76

**Table 6:** The amount (ng) of total and individual heterocyclic aromatic amine contents quantified in 2.5 mL milk (mean ± SD).

Milk Types	IQx	IQ	MeIQx	MeIQ	PhIP	Total	Portion °
Growing up	nd-nq	nd–nq	0.06 ± 0.09 ^e^ (nd–0.24)	nd	0.07 ± 0.11 ^b^ (nd–0.39)	0.13 ± 0.12 ^c^	10.10 ± 9.97 ^c^
Lactose free—semi skimmed	nd	nd–nq	0.35 ± 0.11 ^b^ (0.17–0.46)	nd	nd	0.35 ± 0.11 ^b,c^	27.93 ± 8.64 ^b,c^
Protein-fortified coffee-flavored ^•^	0.05 ± 0.02 ^a^ (0.03–0.06)	0.09 ± 0.01 ^a^ (0.09–0.1)	nd	0.02 ± 0.03 ^b^ (nq–0.05)	0.19 ± 0.02 ^a^ (0.17–0.2)	0.34 ± 0.03 ^b,c^	27.53 ± 2.41 ^b,c^
Protein-fortified cocoa-flavored ^•^	nd-nq	0.01 ± 0.01 ^b^ (nd–0.03)	0.03 ± 0.05 ^e^ (nd–0.1)	0.16 ± 0.16 ^b^ (nq–0.37)	0.02 ± 0.05 ^b^ (nd–0.12)	0.22 ± 0.19 ^b,c^	17.26 ± 15.30 ^b,c^
Protein-fortified vanilla-flavored ^•^	nd	nq	0.23 ± 0.22 ^c,d^ (nd–0.55)	0.43 ± 0.77 ^a^ (nd–1.97)	nd–nq	0.67 ± 0.65 ^a^	53.35 ± 51.79 ^a^
Whole	nd	nd–nq	0.24 ± 0.13 ^c,d^ (nd–0.47)	0.01 ± 0.04 ^b^ (nd–0.12)	nd	0.26 ± 0.13 ^b,c^	20.59 ± 10.73 ^b,c^
Organic	nd	nd–nq	0.41 ± 0.16 ^a^ (0.19–0.49)	nd	nd–nq	0.41 ± 0.16 ^b^	32.48 ± 13.19 ^b^
Skimmed	nd	nd–nq	0.18 ± 0.17 ^d^ (nq–0.41)	nd	nd	0.18 ± 0.17 ^b,c^	14.07 ± 13.35 ^b,c^
Semi skimmed	nd	nd–nq	0.31 ± 0.21 ^b,c^ (nd–0.52)	0.01 ± 0.02 ^b^ (nd–0.06)	nd	0.32 ± 0.21 ^b,c^	25.36 ± 16.84 ^b,c^
Sig.	**	**	**	**	**	**	**

SD: standard deviation; Sig: significance; nd: not detected (below the limit of detection, LOD); nq: not quantified (between LOD and the limit of quantification, LOQ); **: *p* < 0.01; ^a–e^: means with different letters in the same column are significantly different (*p <* 0.05). ^•^: all protein-fortified milk samples were skimmed and lactose-free; °: portion size considered 200 mL of milk (total HAA in 2.5 mL milk × 80). Minimum and maximum values are given between brackets (max–min).

**Table 7:** The total HAA contents of milk samples grouped according to their protein, carbohydrate, and fat contents (mean ± SD).

Milk Groups *	Protein		Carbohydrate		Fat	
	Total HAA	Portion °	Total HAA	Portion °	Total HAA	Portion °
1	0.13 ± 0.04 ^c^	10.44 ± 3.08 ^c^	0.31 ± 0.02 ^a^	24.40 ± 1.93 ^a^	0.37 ± 0.12 ^a^	29.38 ± 9.91 ^a^
2	0.29 ± 0.03 ^b^	23.56 ± 2.76 ^b^	0.35 ± 0.27 ^a^	28.24 ± 21.81 ^a^	0.33 ± 0.11 ^a^	26.22 ± 9.11 ^a^
3	0.42 ± 0.08 ^a^	33.75 ± 6.39 ^a^	0.20 ± 0.12 ^a^	15.79 ± 9.91 ^a^	0.23 ± 0.10 ^a^	18.25 ± 7.99 ^a^
Sig.	**	**	ns	ns	ns	ns

SD: standard deviation; Sig: significance; **: *p* < 0.01; ns: not significant, *p* > 0.05; ^a–c^: means with different letters in the same column are significantly different (*p <* 0.05). *: 1, 2, and 3 stands for low, medium, and high for the protein and carbohydrate contents, and they stand for skimmed, semi-skimmed, and whole milk for the fat contents, respectively; °: portion size considered as 200 mL of milk (total HAA in 2.5 mL milk × 80).

## Data Availability

Data is contained within the article.

## Supplementary Materials

The following are available online at https://www.mdpi.com/article/10.3390/foods10061247/s1, Table S1: The dry matter contents (%) and pH values of UHT milk samples classified according to their types and brands (mean ± SD).
