# Supplementary material for: Is Ultra-High Temperature Processed Milk Safe in Terms of Heterocyclic Aromatic Amines?
Source: Foods. 2021 May 31;10(6):1247. doi: 10.3390/foods10061247 (PMC8227235; doi:10.3390/foods10061247)
Supplement: Supplementary file 1 [file foods-10-01247-s001.zip › foods-1216456-supplementary.pdf]

foods

Table S1. The dry matter contents (%) and pH values of UHT milk samples classified according to their types and brands (mean ± SD).

Type	Brand	n	Dry Matter (%)	pH
Growing up milk	A	3	13.71 ± 0.41	6.88 ± 0.01 [b]
	B	3	14.02 ± 0.05	6.87 ± 0.00 [b]
	C	3	14.16 ± 0.19	6.94 ± 0.06 [a]
	D	3	13.81 ± 0.05	6.97 ± 0.01 [a]
Sig.			ns	**
Lactose free - semi skimmed	B	3	9.59 ± 0.12	6.77 ± 0.01 [a]
	D	3	9.78 ± 0.20	6.71 ± 0.01 [b]
Sig.			ns	**
Protein fortified-coffee flavored milk•	B	3	11.92 ± 0.02	6.82 ± 0.01
Protein fortified-cacao flavored milk•	D	3	10.59 ± 0.39 [b]	6.73 ± 0.02 [a]
	B	3	13.14 ± 0.19 [a]	6.59 ± 0.02 [b]
Sig.			**	**
Protein fortified-vanilla flavored milk•	D	3	11.11 ± 0.05 [b]	6.83 ± 0.02 [a]
	B	3	12.14 ± 0.04 [a]	6.54 ± 0.06 [b]
Sig.			**	**
Whole milk	E	3	10.94 ± 0.03 [d]	6.81 ± 0.00 [a]
	B	3	11.90 ± 0.05 [a]	6.75 ± 0.01 [b]
	D	3	11.26 ± 0.13 [c]	6.75 ± 0.03 [b]
	F	3	10.37 ± 0.06 [e]	6.72 ± 0.01 [b]
	G	3	11.48 ± 0.22 [b]	6.75 ± 0.01 [b]
Sig.			**	**
Organic milk	B	3	11.47 ± 0.16	6.83 ± 0.02 [a]
	D	3	11.67 ± 0.09	6.77 ± 0.00 [b]
Sig.			ns	*
Skimmed milk	B	3	8.87 ± 0.04 [a]	6.71 ± 0.01 [b]
	D	3	8.24 ± 0.12 [b]	6.75 ± 0.01 [a]
Sig.			**	**
Semi skimmed milk	A	3	10.12 ± 0.06 [a]	6.71 ± 0.00 [c]
	H	3	9.87 ± 0.03 [b]	6.71 ± 0.01 [c]
	F	3	9.63 ± 0.10 [c]	6.75 ± 0.01 [b]
	G	3	10.09 ± 0.01 [a]	6.78 ± 0.00 [a]
Sig.			**	**

SD: standard deviation; Sig: significance; **: $p < 0.01$; *: $p < 0.05$; ns: not significant; a-e: means with different letters in the same column and belong to same type of milk are significantly different •: all protein fortified milk samples were skimmed and lactose free.
